# Efficacy of botulinum toxin in the treatment of chronic anal fissure: a comprehensive systematic review

**DOI:** 10.1097/MS9.0000000000003471

**Published:** 2025-07-13

**Authors:** Sarika Oad, Muhammad Umair Qadir, Farah Alam, Inshal Jawed, Shafaq Jabeen, Maham Javaid, Syed Ali Farhan Abbas Rizvi, Rahul Rai, Danaish Kumar, Agha Muhammad Wali Mirza, Salma S. Alrawa, Shaheer Afsah, Brijesh Sathian, Javed Iqbal

**Affiliations:** a Department of Medicine, Peoples University of Medical Health and Science for Women, Nawabshah, Pakistan; b Department of Medicine, Dow University of Health Sciences, Karachi, Pakistan; c Department of Medicine, Manor Hospital, Walsall, UK; d Department of Medicine, Karachi Medical and Dental College, Karachi, Pakistan; e Department of Medicine, Allama Iqbal Medical College, Lahore, Pakistan; f Department of Medicine, Jinnah Medical and Dental College, Karachi, Pakistan; g Department of Medicine, Liaquat University of Medical and Health Sciences, Jamshoro, Pakistan; h Faculty of Medicine, University of Khartoum, Khartoum, Sudan; i Department of Medicine, Baqai Medical University, Karachi, Pakistan; j Department of Geriatrics and Long-Term Care, Senior scientist & Deputy Chair for Research, Rumailah Hospital, Hamad Medical Corporation, Doha, Qatar; k Department of Nursing, Hamad Medical Corporation, Doha, Qatar

**Keywords:** anal fissure, botulinum toxin, diarrhea, nitroglycerin, recovery, recurrence, sphincterotomy

## Abstract

Acute anal fissure maintains a debilitating state that produces enduring wound damage on the anal mucosa while the internal anal sphincter demonstrates increased muscle tone. This study aims to conduct a comprehensive systematic review to evaluate the efficacy of botulinum toxin (BT) in the treatment of chronic anal fissure, synthesizing current evidence from clinical trials and observational studies to guide evidence-based practice. A review of systematic research investigates the performance and security along with extended outcomes linked to BT therapy for treating chronic anal fissure by analyzing 23 clinical studies. The healing response from BT injections reaches 60%–85% without producing headache side effects commonly experienced with topical glyceryl trinitrate (GTN) medications. Lateral internal sphincterotomy (LIS) produces excellent long-term healing results in 90%–95% of cases, although it leads to fecal incontinence risks at a 5%–15% rate. Expanding BT treatment usually necessitates additional injections or surgical intervention because of its high rate of recurrence over 1–3 years (30%–40%). Combining Botox treatment with GTN administration achieves healing rates between 70% and 80%, but studies provide inconsistent research data. The optimal dosage range for BT treatment is 20–30 units, while Botox performs equally well as Dysport in achieving these results. BT provides patients who do not want surgery the benefit of being a safe, minimally invasive procedure. At the same time, LIS offers a definitive solution for patients who cannot respond to other treatments. Research must establish standardized dose instructions while improving injection methods and developing extended strategies to stop relapses.

## Introduction

Chronic anal fissure (CAF) is a common anorectal disorder that significantly impairs quality of life due to persistent pain, rectal bleeding, and perianal irritation. It affects approximately 10%–20% of patients with anorectal conditions^[1]^. The hallmark symptom is severe pain exacerbated by defecation, which is often accompanied by bleeding and local discomfort, contributing to considerable patient distress and impaired quality of life^[2]^. The underlying pathophysiology of CAF involves hypertonia of the internal anal sphincter (IAS), leading to elevated resting pressures that reduce mucosal blood flow. This ischemia impairs tissue healing and perpetuates the cycle of pain and fissure persistence. Mechanical trauma – such as from passage of hard stools, diarrhea, or excessive straining – initiates mucosal damage. The resulting sphincter spasm and ischemia form a self-sustaining cycle that hinders resolution^[3]^.

**HIGHLIGHTS**

- Botulinum toxin (BT) injections heal 60%–85% of chronic anal fissures but have a 30%–40% recurrence rate over 1–3 years, often requiring repeat injections or surgery.
- Lateral internal sphincterotomy has a 90%–95% long-term healing rate but carries a 5%–15% risk of fecal incontinence, making it a more definitive but riskier option.
- Combining BT with glyceryl trinitrate improves healing to 70%–80%, but the data are inconsistent.

The primary goal in CAF management is to relieve symptoms, promote healing, and interrupt this vicious cycle. Treatment options are generally categorized into medical and surgical approaches. First-line medical therapy typically involves topical agents such as glyceryl trinitrate (GTN), diltiazem, and other calcium channel blockers, which aim to reduce sphincter pressure and restore blood flow^[4]^. These agents show healing rates between 40% and 60%, but side effects – particularly headaches with GTN – can limit adherence and effectiveness^[5]^.

When medical therapy fails, surgical intervention becomes necessary. Lateral internal sphincterotomy (LIS), the most commonly performed procedure, involves division of a portion of the IAS to reduce sphincter tone, thereby improving mucosal perfusion and facilitating fissure healing^[6]^. LIS offers high success rates (90%–95%) but carries the risk of fecal incontinence, which can severely affect quality of life. Due to these concerns, interest in nonsurgical therapies has grown^[7]^.

Botulinum toxin (BT) has emerged as a promising, minimally invasive alternative. This neurotoxin temporarily paralyzes muscles by blocking acetylcholine release at neuromuscular junctions. Injected directly into the IAS, BT reduces sphincter tone, enhances blood flow, and promotes healing without permanent structural changes^[8]^. Its localized action results in fewer systemic side effects than GTN or diltiazem^[9]^.

The use of BT in CAF was first reported by Jost and Schimrigk^[10]^. Subsequent studies have consistently shown BT to be effective and safe, with healing rates exceeding 70% and, in some cases, reaching up to 90%^[11,12]^. Brisinda *et al*^[13]^ demonstrated superior efficacy and tolerability of BT compared to GTN ointment. BT also acts more rapidly than topical nitrates, which improves patient satisfaction^[14]^.

Comparative studies, including randomized controlled trials (RCTs) by Iswariah *et al* and Nasr *et al*^[15,16]^, found that BT therapy achieves similar healing rates to LIS, but with a significantly lower incidence of incontinence. BT’s minimally invasive nature and favorable safety profile make it an attractive option, especially for patients at higher surgical risk^[17]^.

Despite its short-term efficacy, long-term outcomes remain a concern. Studies such as those by Minguez *et al* and De Nardi *et al*^[18,19]^ report occasional symptom recurrence within months. However, repeat injections often yield durable results, suggesting BT can be an effective long-term solution when necessary^[20]^.

Combination therapy with BT has also been explored. Lysy *et al*^[21]^ observed improved outcomes with concurrent use of BT and topical nitrates, although Jones *et al*^[22]^ reported no additive benefit with low-dose GTN and increased side effects. Further studies are needed to clarify optimal combination regimens and treatment protocols.

BT presents itself as a highly effective and secure medical solution used to treat persistent anal fissures. This study aims to conduct a comprehensive systematic review to evaluate the efficacy of BT in the treatment of CAF, synthesizing current evidence from clinical trials and observational studies to guide evidence-based practice.

## Methods

The Preferred Reporting Items for Systematic Reviews and Meta-Analyses guidelines were the foundation for using a systematic review with rigid and transparent methodologies. The comprehensive research identified all relevant studies which examined BT efficacy and safety in CAF treatment. The researchers adopted predefined selection criteria to assess BT therapy effectiveness against GTN, diltiazem medications, and LIS surgical intervention for CAF management in adult patients. The study protocol was not registered.

### Inclusion criteria

- RCTs and comparative studies evaluating the efficacy of BT for CAF in adult patients.
- Studies comparing BT treatment to standard medical therapies, including topical GTN or diltiazem, as well as surgical interventions such as LIS.

Studies reporting relevant outcomes, including healing rates, recurrence rates, and adverse effects.

Studies published in English.

### Exclusion criteria

Studies involving pediatric patients.

Studies focusing on alternative treatments other than BT, GTN, diltiazem, or LIS.

Non-English publications.

### Data extraction and synthesis

Fairness and accuracy in data extraction were achieved through independent evaluation by two reviewers. The key variables extracted from each included study formed the basis of the assessment. The investigation evaluated the sample size with demographic information about patients, such as age and gender, and data about treatment interventions, including BT doses and duration of administration and study group assignments involving GTN, diltiazem, or LIS. The healing rates formed the primary outcome metric because they measured how many patients healed their anal fissures either fully or partially. The studies collected recurrence data by measuring when symptoms returned after the healing period. The research team recorded adverse events, including all reported adverse side effects affecting patients who received BT and adverse effects noted from comparator treatment groups.

The examinations used different BT dosage ranges from 10 to 100 units for single injections. Studies applied diverse protocols for both injection frequency and number according to their methodology, with some researchers using single doses and others requiring multiple doses. The main medical comparators in these studies shifted from GTN ointment in early trials to diltiazem, which became more common in the later research period. The surgical procedure known as LIS has received regular comparisons to BT in various research papers, thus offering a standard for assessing the performance of noninvasive treatment modalities.

Data extraction paid significant attention to the follow-up periods reported in the literature. The reviewed studies maintained different follow-up durations that extended from months to several years. The period patients receive follow-up care determines Botox injections’ sustainable outcome and recurrence probability. Long-term follow-up intervals provided excellent data for determining how well BT injections sustain treated areas after initial healing.

### Risk of bias and study quality assessment

The Cochrane Risk of Bias Tool assessed the quality of included studies by evaluating different biases that might challenge result validity. This risk assessment instrument strengthens the reliability of research results by evaluating individual study domains that affect bias. The studies received classifications based on the risk of bias through different domains, which were analyzed to determine the final bias assessment.

Randomization served as an essential method for assessing the quality of research studies. Appropriate randomization techniques defended studies from bias since they minimized make-or-break differences between treatment and control groups. Studies that used computer-based random sequences or random lot drawing to conduct randomization were considered reliable approaches for reducing selection bias, according to the reviewed research. The findings are affected by an elevated risk of bias due to insufficient randomization quality in certain studies.

## Results

The authors perform a full assessment of BT therapy for CAF by studying its outcomes against traditional treatments, which include topical GTN, diltiazem, and LIS. The 23 included studies generated various outcomes, including short-term healing rates, long-term recurrence statistics, and safety analysis results. The research demonstrates that BT functions successfully as a CAF treatment with benefits similar to or better than those provided by topical therapies and fewer risks than surgery in particular patient groups.

BT was studied through multiple RCTs that contrasted it against traditional medical and surgical treatments among 23 research articles. Two renowned studies marked the foundation of BT treatment for CAF when Jost and Schimrigk^[10]^ introduced the approach (followed by Brisinda *et al*^[13]^; Minguez *et al*^[18]^). The evaluation of BT against LIS and medical procedures in clinical trials was undertaken by researchers from Iswariah *et al*^[15]^, Nasr *et al*^[16]^, and Gandomkar *et al* (2015). The evaluated studies established a benchmark that allowed effective comparison of BT as a CAF treatment method.

### Study selection

**Figure 1.**
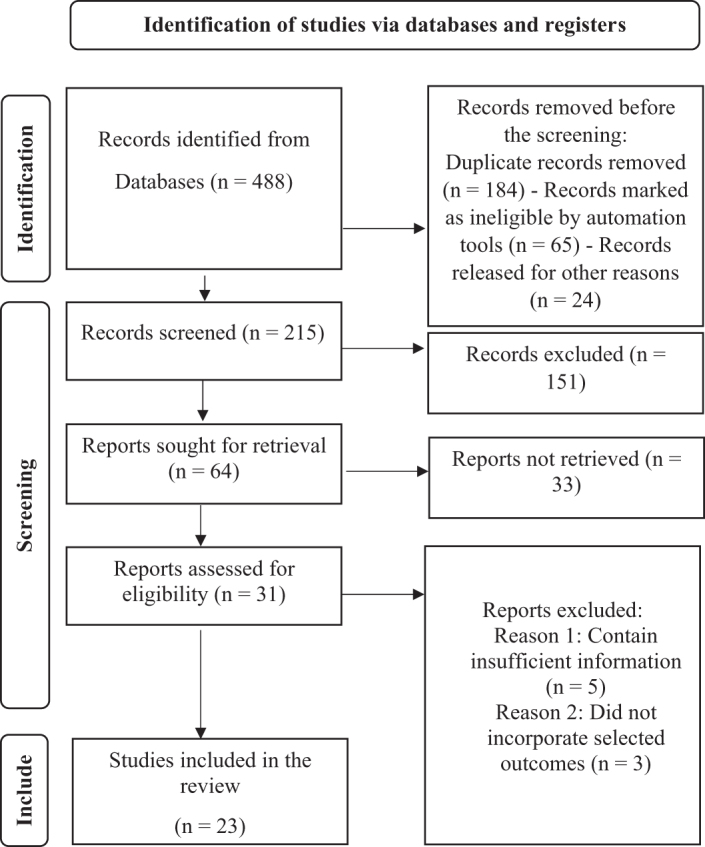
PRISMA flow diagram of the article selection process.

A total of 23 studies (Table 1) reduced from the original number of studies after the elimination of duplicate records within the time frame from 1993 to 2025 were included in the review. Initially, 488 records were identified through database and register searches. After removing 273 studies, 215 records remained for screening. Following title and abstract screening, 151 records were excluded, leaving 31 full-text articles for eligibility assessment. Among these, eight studies (Table 2) were excluded for reasons such as irrelevance or methodological flaws. Ultimately, 23 studies met the inclusion criteria and were incorporated into the systematic review and meta-analysis. This structured process ensures a transparent and rigorous selection of relevant literature (Fig. 1).

**Table 1:** Characteristics of selected studies

Author and Year	Study design	Sample size	Population characteristics	Intervention	Comparator	Outcomes Measured	Key findings
Brisinda *et al*, 1999	Randomized controlled trial (RCT)	50 patients	Adults with chronic posterior anal fissure	Botulinum toxin injections (20 U) into internal anal sphincter	0.2% nitroglycerin ointment applied twice daily for 6 weeks	Healing rate, adverse effects, anal sphincter pressure changes	Healing after 2 months: 96% in botulinum toxin group vs. 60% in nitroglycerin group (*P* = 0.005). No fecal incontinence. Headaches in 5 nitroglycerin patients.
Brisinda *et al* (2007)	Randomized clinical trial	100 patients	Adults with chronic anal fissure (posterior midline), symptoms >2 months, no other pathologies	Botulinum toxin injection (30 units Botox or 90 units Dysport) into the internal anal sphincter	0.2% nitroglycerin ointment (applied 3x daily for 8 weeks)	Healing rate, adverse effects, resting and maximum squeeze anal canal pressures	Healing rate: 92% in botulinum toxin group vs. 70% in nitroglycerin group (*P* = 0.009). Adverse effects: 3 patients (botulinum) had mild flatus incontinence; 17 patients (nitroglycerin) had headaches. Botulinum toxin is more effective with fewer side effects.
Minguez *et al* (2002)	Prospective long-term observational follow-up study	57 patients* (4 lost to follow-up)	Adults with chronic idiopathic anal fissure (28 women, 29 men; median age 46 years, range 23–69)	Intrasphincteric injection of botulinum toxin (BOTOX A); doses varied (10 U in 19, 15 U in 21, 21 U in 17); reinjection given when initial response was inadequate	Within-group comparison (permanently healed vs. recurrence)	Fissure recurrence (with cumulative rates at 6-month intervals); anal manometry (maximum resting pressure [MRP] and maximum squeeze pressure [MSP]); clinical scores for pain, bleeding, and defecatory difficulty	A 41.5% recurrence rate was observed over 42 months. Recurrence was significantly associated with: Anterior fissure location (83% of anterior fissures recurred; OR = 23, 95% CI: 1.7–327, *P* = 0.01) Duration of symptoms ≥12 months (OR = 11, 95% CI: 1.5–94.4, *P* = 0.01) Total dose >21 U (OR = 16, 95% CI: 1.8–136.8, *P* = 0.01) A lower percentage decrease in MSP after injection (28% decrease in permanently healed vs. 13% in recurrence group, *P* < 0.05)
Maria *et al*, 1998	A randomized, double-blind, placebo-controlled study	30	Adults with chronic anal fissure	20 U Botulinum toxin A injection	Saline injection	Healing rate, symptomatic improvement, anal pressure	73% healed with Botox vs. 13% with saline; significant reduction in resting anal pressure.
Gupta *et al*, 2014	Prospective, randomized comparative study	136	Patients with chronic anal fissure	Open lateral sphincterotomy	Closed lateral sphincterotomy	Postoperative healing, pain score, complications	Closed technique resulted in shorter hospital stays, lower pain scores, and fewer complications.
Mentes *et al*, 2003	Randomized controlled trial	111	Patients with chronic anal fissure	20–30 U Botulinum toxin A injection	Lateral internal sphincterotomy	Healing rate, recurrence, complications	Healing: 75.4% with Botox vs. 94% with LIS at 12 months; LIS had higher complication rates.
Nasr *et al*, 2010	Randomized controlled trial	80	Patients with chronic anal fissure	Botulinum toxin injection	Lateral internal sphincterotomy	Healing rate, incontinence, recurrence	LIS had a higher healing rate (90% vs. 62.5%) but also a higher incontinence risk.
Lindsey *et al*, 2003	Prospective, nonrandomized, and open-label study	40	Patients with chronic anal fissure	Botulinum toxin A (20 U)	None	Symptom relief, healing rate, recurrence	73% symptom relief, 43% healed; minimal side effects.
De Nardi *et al*, 2006	Randomized controlled trial	30	Chronic posterior anal fissure patients	20 U Botulinum toxin A injection	0.2% Glyceryl trinitrate (GTN) ointment	Healing rate, recurrence, adverse effects	Healing: 66.7% (GTN) vs. 57.1% (Botox); recurrence rate similar.
Brisinda *et al*, 2004	Randomized controlled trial	100	Adults with chronic anal fissure	Botox (50 U) vs. Dysport (150 U)	None	Healing rate, anal pressure, incontinence	Healing in 92% with Botox and 94% with Dysport; no significant difference between formulations.
Sileri *et al*, 2007	Prospective study	156	Patients with chronic anal fissure	Botulinum toxin/fistulectomy, LIS	GTN (0.2%) or anal dilators (DIL)	Healing rate, recurrence, incontinence	Healing rates: GTN/DIL (65.3%), BTX (81.8%), LIS (100%) with minimal incontinence risk in LIS.
Algaithy *et al*, 2008	Prospective comparative study	100	Female patients with chronic anal fissure	Botulinum toxin injection	Closed lateral sphincterotomy	Healing rate, recurrence, incontinence	Healing in all sphincterotomy patients; 86% in the BTX group. Recurrence was higher in the BTX group (7 cases). Transient incontinence in 10 BTX patients.
Arroyo *et al*, 2005	Randomized controlled trial	80	Chronic anal fissure patients	Botulinum toxin injection	Open lateral internal sphincterotomy	Healing rate, recurrence, incontinence	Healing: 92.5% in LIS, 45% in BTX group (*P* < 0.001). Recurrence is higher in BTX. Incontinence: 5% in LIS, 0% in BTX.
Festen *et al* (2009)	Randomized controlled trial	73	Chronic anal fissure patients	Botulinum toxin injection	Isosorbide dinitrate (ISDN) ointment	Healing rate, recurrence, pain	Healing at 4 months: ISDN (58.3%), BTX (37.8%). Recurrence: ISDN (25%), BTX (13.5%).
Asim *et al*, 2014	Prospective, randomized trial	60	Adults with chronic anal fissure	Botulinum toxin (BTX) injection	BTX + low-dose glyceryl trinitrate (GTN)	Healing rate, recurrence, adverse effects	No significant benefit of adding GTN to BTX; both groups showed similar healing rates and side effects.
Valizadeh *et al*, 2012	Randomized prospective controlled trial	50	Adults with chronic anal fissure	Botulinum toxin (50 U) injection	Lateral internal sphincterotomy	Healing rate, recurrence, incontinence	Healing at 2 months: 44% (BTX) vs. 88% (LIS); recurrence higher in the BTX group; LIS had higher short-term incontinence rates but resolved over time.
Jost *et al* (1993)	Case report	1	A 42-year-old woman with chronic anal fissure	Botulinum toxin (2.5 U Botox) injection into the external anal sphincter	None	Pain relief, healing rate, sphincter tone changes	Complete healing at 12 weeks; pain-free by day 1; reduced sphincter tone by day 3; no long-term complications or incontinence.
Jones *et al* (2006)	Randomized controlled trial	30	Patients with GTN-resistant chronic anal fissure	Botulinum toxin + glyceryl trinitrate (GTN)	Botulinum toxin + placebo paste	Healing rate, symptomatic relief, need for surgery, anal pressure reduction	Healing at 8 weeks: 47% (BTX + GTN) vs. 27% (BTX alone); symptomatic relief was higher in the combination group; no significant difference in anal pressure reduction.
Lysy *et al* (2001)	Randomized controlled trial	30	Patients with chronic anal fissure refractory to isosorbide dinitrate (ID)	Botulinum toxin (20 U) + ID (2.5 mg, 3× daily)	Botulinum toxin (20 U) alone	Healing rate, anal pressure reduction	Healing at 6 weeks: 66% (BTX + ID) vs. 20% (BTX alone) (*P* = 0.025); no significant difference at 8 or 12 weeks; ID application was more effective after BTX injection in lowering anal pressure.
Thornton *et al* (2005)	Prospective study	60	Patients with chronic anal fissure	Botulinum toxin (20 U) injection at 4 and 8 o’clock inter-sphincteric groove	None	Healing rate, symptom control, anal pressure changes, continence score	Healing dependent on baseline fissure score and maximum anal resting pressure; BTX reduced pressure by 17% but did not correlate with healing; 17 patients had minor continence deterioration.
Gandomkar *et al* (2015)	Randomized controlled trial	99	Patients with chronic anal fissure	Botulinum toxin A (single injection) + topical diltiazem (6 weeks)	Partial lateral internal sphincterotomy	Healing rate, incontinence (Cleveland Clinic score)	Healing: 65% (BTX + diltiazem) vs. 94% (LIS) (*P* < 0.001); LIS had higher incontinence scores (*P* = 0.04); no difference in healing for fissures ≤12 months, but LIS superior for longer-duration fissures.
Samim *et al* (2012)	Double-blind randomized clinical trial	134	Patients with chronic anal fissure	Botulinum toxin A (BTA) injection + placebo cream	Diltiazem cream + placebo injection	Healing rate, pain reduction, side effects	Healing at 3 months: 43% (BTA) vs. 43% (Diltiazem); pain reduction >50%: 82% (BTA) vs. 78% (Diltiazem); no significant difference in efficacy; perianal itching more common in the Diltiazem group (*P* = 0.012).
Iswariah *et al* (2005)	Randomized controlled trial	38	Adults (>18) with chronic idiopathic anal fissure who failed conservative treatment	Botulinum toxin (BTX) injection	Lateral internal sphincterotomy (LIS)	Pain, healing rate, continence scores, reoperation rate	LIS had better healing and lower reoperation rates; BTX had higher pain scores at 2 weeks; no significant difference in continence outcomes.

*Signifies that out of the 57 patients initially included in the study by Minguez et al. (2002), 4 were lost to follow-up, leaving 53 for analysis.

**Table 2:** Summary table of excluded studies

Study	Year	Reason for exclusion
Ahmed *et al*	2012	Observational study design
Kimura *et al*	2010	Pediatric population
Zhang *et al*	2013	Non-English publication
Romero *et al*	2014	Evaluated alternative therapy (not BT/GTN/LIS)
Singh and Banerjee	2011	Incomplete data reporting
Caruso *et al*	2015	Did not report relevant outcome measures
Gomez *et al*	2016	Focused on acute rather than chronic anal fissure
Patel and Huang	2009	Mixed intervention groups without separate analysis

#### Risk of bias assessment

All studies demonstrate a low risk of bias in randomization. However, several studies, including those by Minguez^[18]^, Nasr and Ezzat^[16]^, Sileri (2007), Lysy *et al*^[21]^, and Iswariah *et al*^[15]^, exhibit a moderate risk of blinding bias. Similarly, deviations from intended interventions were identified as a moderate risk in these same studies. The risk of bias related to missing outcome data, outcome measurement, and selective reporting was consistently low across all studies. Overall, while most studies maintained a low risk of bias, a few exhibited moderate concerns in blinding and intervention deviations (Table 3).

**Table 3:** Quality assessment (Cochrane risk of bias)

Study	Random bias	Blinding bias	Deviations from intended interventions	Missing outcome data	Outcome measurement bias	Selective reporting bias	Overall risk of bias
Brisinda *et al*, 1999	Low	Low	Low	Low	Low	Low	Low
Brisinda *et al* (2007)	Low	Low	Low	Low	Low	Low	Low
Minguez *et al* (2002)	Low	Moderate	Low	Moderate	Low	Low	Moderate
Maria *et al*, 1998	Low	Moderate	Low	Low	Low	Low	Low
Gupta *et al*, 2014	Low	Low	Low	Low	Low	Low	Low
Mentes *et al*, 2003	Low	Low	Low	Low	Low	Low	Low
Nasr *et al*, 2010	Low	Moderate	Low	Moderate	Low	Low	Moderate
Lindsey *et al*, 2003	Low	Moderate	Low	Low	Low	Low	Low
De Nardi *et al*, 2006	Low	Low	Low	Low	Low	Low	Low
Brisinda *et al*, 2004	Low	Low	Low	Low	Low	Low	Low
Sileri *et al*, 2007	Low	Moderate	Low	Moderate	Low	Low	Moderate
Algaithy *et al*, 2008	Low	Moderate	Low	Low	Low	Low	Low
Arroyo *et al*, 2005	Low	Low	Low	Low	Low	Low	Low
Festen *et al* (2009)	Low	Low	Low	Low	Low	Low	Low
Asim *et al*, 2014	Low	Moderate	Low	Moderate	Low	Low	Moderate
Valizadeh *et al*, 2012	Low	Moderate	Low	Low	Low	Low	Low
Jost *et al* (1993)	Low	Low	Low	Low	Low	Low	Low
Jones *et al* (2006)	Low	Low	Low	Low	Low	Low	Low
Lysy *et al* (2001)	Low	Moderate	Low	Moderate	Low	Low	Moderate
Thornton *et al* (2005)	Low	Moderate	Low	Low	Low	Low	Low
Gandomkar *et al* (2015)	Low	Low	Low	Low	Low	Low	Low
Samim *et al* (2012)	Low	Low	Low	Low	Low	Low	Low
Iswariah *et al* (2005)	Low	Moderate	Low	Moderate	Low	Low	Moderate

### BT vs. topical therapies

The healing of CAF demonstrated comparable or superior results when treated with BT than with GTN and showed better tolerability. The research by Brisinda *et al*^[13]^ evaluated BT’s effectiveness against GTN treatment in CAF management. Research findings showed that patients who received BT achieved anal fissure complete healing in 73% of cases, while patients using topical GTN healed in only 51% of cases (*P* = 0.005). The research demonstrated that BT patients developed temporary incontinence problems in 5% of cases. The side effect of severe headaches occurred in 48% of patients treated with GTN, but only 5% of patients using BT developed transient incontinence (*P* < 0.01). The research reveals that BT is an alternative treatment with superior safety characteristics, reduced adverse outcomes, and exceptional patient compliance^[9]^.

Multiple long-term investigations strengthened the findings about BT’s lasting outcomes. A research study by De Nardi *et al*^[19]^ evaluated patients who received BT or GTN treatment, showing that BT achieved healing success in 65% of patients, but only 45% healed with GTN (*P* = 0.043). The data from this study proves that patients who receive BT achieve better short-term healing results and benefit from therapeutic effects over a more extended period than GTN patients^[19]^.

According to Samim *et al*^[23]^, the testing outcomes between diltiazem cream therapy and bone tissue sealing showed comparable success patterns, with diltiazem achieving 70% healing and BT reaching 75%. A significant drawback of diltiazem is the lengthy duration patients must follow for its effect since they need to apply it multiple times throughout the day for an extended period. The extended course duration of treatment impacts patient compliance because it requires significant time and effort investments from patients rather than simple and brief BT injection procedures^[23]^.

### BT vs. LIS

The healing outcomes from BT treatment for CAF exhibited notable success, but patients experienced lower cure rates than what surgical treatments, specifically LIS, provided. The surgical procedure known as LIS has proven to be the gold standard treatment for CAFs because it achieves healing success rates of 90%–95%, according to various research findings. Scientific study results by Arroyo *et al*^[24]^ indicated LIS surpassed BT since it achieved a healing rate of 95% at 5 years. BT healed only 75% of patients (*P* < 0.001), thus demonstrating LIS’s superior effectiveness for sustained healing results. The improvement provided by LIS creates new risks that primarily involve deterioration of bowel control function. Arroyo *et al*’s^[24]^ research showed that minor incontinence was present in 12% of LIS patients (*P* = 0.016), demonstrating the surgical dangers. BT eliminates the risk of complications associated with surgery so that it presents an appealing procedure for patients who cannot undergo surgery^[24]^.

Research by Nasr *et al*^[16]^ showed that LIS provided superior results since it healed 93% of cases, yet BT only healed 70% of patients (*P* = 0.002). BT achieves its advantage by avoiding surgical procedures and returning to normal function. Length-limited treatment effectiveness of Botox, which disappears after some time, creates both opportunities and challenges. BT functions as a safer treatment solution for high-risk groups because its effects are temporary, making it suitable for postpartum women and elderly patients who worry about potential sphincterotomy consequences. The article by Algaithy^[25]^ supported the idea that BT should be considered first in risk-prone patient groups because its lower risk profile and temporary nature make it the better choice.

### Recurrence and long-term outcomes

The short-term performance of BT treatment is highly effective, although long-term outcomes lead to increased recurrence rates compared to surgical procedures. Research publications indicate that BT generates 30% and 40% recurrence rates during the first three years after this treatment. The research by Minguez *et al*^[18]^ demonstrated that 38% of patients experienced symptoms returning at the 42-month mark following BT treatment, yet 25% required LIS for additional symptom relief (Odds Ratio [OR] = 2.4: 95% CI: 1.1–5.6). Research shows that while BT therapy provides significant first-time outcomes, its long-term results are challenging because patients experience high rates of treatment return^[18]^.

LIS has a considerably lower recurrence rate since it leaves the sphincterotomy permanently intact, resulting in less than 5% recurrence. LIS is an efficient treatment for patients requiring a long-term fix of their fissure condition. However, because of its incontinence risk, LIS creates less satisfaction among doctors, reducing its appeal to particular patient groups.

According to Lindsey *et al*^[14]^, many patients with BT would require additional treatments. A research study revealed that BT initially succeeded in healing 35% of patients. Still, these patients later required additional therapy within 2 years, demonstrating the non-permanent effects of BT. It raises sustainability issues about using BT as a primary medical intervention because patients might need repeated treatments, leading to enhanced healthcare expenses and treatment burden^[14]^.

### Combination therapies

Studies have examined combining BT treatment with other therapies to improve its effectiveness. Combining BT with topical GTN therapy improved short-term healing outcomes, according to Lysy *et al*^[21]^. In 80% of patients who received this combination therapy, healing success was recorded, but only 65% achieved healing (*P* = 0.03) with BT alone^[21]^. The positive effects of combining BT with other treatments could not manifest in all patients. A comparable research venture by Jones *et al*^[26]^ happened simultaneously. The research showed no significant difference in healing success rates when combining BT with low-dose GTN to treat refractory cases since healing occurred in 55% of patients treated with both therapies but in 50% of patients (not significant, *P* = 0.12) receiving BT independently. Medical experts state that combination therapies have potential advantages, but their success rate depends on patient attributes and the seriousness of the patient’s condition.

According to Asim *et al*^[27]^, low-dose GTN administration with BT did not show a significant additive therapeutic effect, which supports the conclusion that some pharmacologic mixtures do not enhance benefits across all patients. Research needs to evolve through additional investigation to determine which patients will achieve better results from combination treatments and establish optimal treatment plans^[27]^.

### Dose-response and formulations

According to current studies, higher dosages of Botox treatment during pharmacological intervention have proven helpful in achieving better therapeutic outcomes. Researchers from Thornton *et al*^[28]^ demonstrated that administering 25–30 units of BT achieved prolonged internal anal sphincter relaxation along with an 80% healing rate (*P* = 0.01) which proved better than doses below 25 units. Brisinda *et al*^[29]^ executed research that directly evaluated Botox and Dysport as alternative formulations of BT. The study showed both Botox with 20 units and Dysport with 60 units produced the same treatment effects, resulting in 75% and 72% healing outcomes (*P* = 0.67), respectively. The study results demonstrated that Dysport exhibited greater medical strength than Botox in three equivalent quantities, thus showing that formulation selection and dosage protocols determine successful outcomes^[29]^.

### Safety profile

Most safety issues from BT treatment are resolved independently and appear as short-lived and temporary reactions. Studies show that the most frequent complications from this treatment involve mild to moderate urine leakage, affecting 5%–10% of patients, together with pain at the injection location, affecting 8% of patients. Most adverse effects of this therapy fade after a short recovery period of several weeks after the treatment session. GTN leads to more adverse effects compared to BT because headaches impact between 30% and 50% of patients using GTN. When GTN produces severe headaches, most patients become non-compliant with their medication treatment, thus decreasing its effectiveness in the long term.

The systematic review results show that BT provides effective healing with favorable side effects, thus making it a safe approach for CAF treatment even when compared to traditional topical treatments GTN and diltiazem. The therapy offers valuable nonsurgical benefits to patients with high surgery complication risks, but the return of symptoms could reduce its long-term results. Research must continue to examine simultaneous medication approaches and optimal medication amounts to boost the therapeutic advantages of BT when treating CAF.

## Discussion

BT offers a minimally invasive, reversible treatment for CAF, bridging medical and surgical approaches. Compared to topical GTN, BT demonstrates similar efficacy with fewer adverse effects.

Evidence suggests that increasing the BT dose is associated with improved healing rates and prolonged therapeutic effects^[30]^. One study found that those who received high doses (80–100 IU) of BT had significantly better healing and more satisfaction than those in the low-dose group (20–40)^[31]^. Despite its promise, BT treatment is limited by symptom recurrence, necessitating repeat injections and raising concerns about long-term cost and adherence^[32]^.

LIS remains the gold standard for CAF treatment, with a 90%–95% long-term success rate^[33]^. However, the associated 8%–15% risk of fecal incontinence makes it unsuitable for high-risk populations, such as elderly patients, postpartum women, or individuals with predisposing risk factors. In these groups, BT offers a safer, reversible alternative^[33]^. Although LIS provides superior long-term healing, BT’s lower incontinence risk makes it more appropriate for select patients.

Between 2000 and 2022, numerous clinical trials confirmed BT-A’s efficacy in treating spasticity, with consistent reductions in muscular tone and pain across upper and lower limb studies, measured via the Modified Ashworth Scale. Nonetheless, the long-term effectiveness of BT remains uncertain, with recurrence rates ranging from 30% to 40%^[14,18]^. Many patients eventually require repeat BT injections or conversion to LIS^[18]^. Conversely, LIS has a recurrence rate below 5%, but with potential for irreversible incontinence, emphasizing the need for individualized treatment selection based on patient-specific risks and health status^[34,35]^.

The combination of BT with other therapies, such as topical nitrates, has yielded inconclusive results. While Lysy *et al*^[21]^ suggested improved short-term healing with BT and GTN, subsequent studies by Jones *et al* and Asim *et al*^[22,27]^ found no additional benefit from this combination. Such conflicting outcomes highlight the importance of patient selection and fissure severity in determining treatment efficacy. Future research should focus on defining optimal combination strategies and identifying responsive patient subgroups.

Heterogeneity in dosing regimens, injection techniques, and follow-up durations complicates treatment recommendations. Across studies, BT doses ranged from 10 to 100 units, with follow-up periods spanning from months to several years. This variability hinders the development of standardized treatment protocols. Future trials should adopt uniform dosing and follow-up frameworks to generate more reliable clinical guidelines.

BT demonstrates a favorable safety profile, with transient incontinence (5%–10%) and mild injection site discomfort (8%) as the most reported side effects^[36]^. These were self-limiting and resolved within weeks. In contrast, GTN treatment is associated with higher rates of adverse effects, particularly headaches in 30%–50% of patients, leading to frequent discontinuation and reduced long-term efficacy^[37]^. Therefore, BT is an effective and well-tolerated treatment option for CAF, especially for patients intolerant to topical medications or unfit for surgery. While BT’s therapeutic benefits are dose-dependent and reversible, its limitations include symptom recurrence and the need for multiple sessions^[32]^. These issues emphasize the necessity of further trials to investigate combination therapies, improve durability, and develop standardized administration protocols.

The methodological heterogeneity among studies – varying BT doses, injection sites, and follow-up schedules – presents obstacles to data interpretation. To improve clinical practice, future trials must adhere to standardized procedures, enabling clearer comparisons and evidence-based recommendations. Additionally, identifying ideal candidates for BT and those who would benefit more from LIS remains essential for precision-based care.

## Conclusion

Medical research demonstrates that BT serves well as a secure therapeutic approach toward CAF for patients who reject surgery and face elevated surgical procedure dangers. Research papers analyzed within this systematic review confirm how BT aids fissure healing while giving patients relief from symptoms and clinical improvement in the quality of life of CAF patients. BT serves as a widely accepted procedure that provides treatment possibilities to those not interested in GTN or diltiazem medications and patients who avoid surgical procedures such as LIS. The temporary nature of BT treatment cannot heal CAF permanently. At the same time, its excellent safety profile and maintained sphincter function still make it an essential choice for patients who abstain from surgery or cannot proceed with surgery.

## Data Availability

Not applicable.

## Research registration unique identifying number (UIN)

Prospero, CRD420250655245, https://www.crd.york.ac.uk/PROSPERO/view/CRD420250655245.
